# Editorial: Women in psychiatry 2022: Psychopharmacology

**DOI:** 10.3389/fpsyt.2022.1126021

**Published:** 2023-01-10

**Authors:** Rosana Camarini, Alfreda Stadlin, Reem Kais Jan

**Affiliations:** ^1^Department of Pharmacology, Institute of Biomedical Sciences, University of São Paulo, São Paulo, Brazil; ^2^College of Medicine, Ajman University, Ajman, United Arab Emirates; ^3^College of Medicine, Mohammed Bin Rashid University of Medicine and Health Sciences, Dubai, United Arab Emirates

**Keywords:** women, cocaine, clozapine, antipsychotic, epigenetics, escitalopram, D3 receptor antagonist, Akt

Still a male-dominated field, science has often undervalued the work of women. We are not equally represented at higher career levels and face more professional obstacles than our male counterparts. One among many examples, Rosalind Franklin's name is the first that come to mind. In the discovery of the DNA double helix, despite her crucial X-ray diffraction work, she never got the same recognition as her male counterparts Watson and Crick (1).

Only 12 women have won the Nobel Prize in Physiology or Medicine since 1901. The first one was Gerty Cori, in 1947, for elucidating pathways of glucose metabolism. She shared the award with her husband, Carl Cori. Albeit working together, her salary was much lower than his ^1^.

One of the most renowned scientists in history, neurobiologist Rita Levi-Montalcini had to deal with discrimination against women in science, widespread antisemitism and a father who was not supportive of women's education. Despite all these challenges, she won a Nobel Prize in 1986, with Stanley Cohen, for their discoveries of growth factors ^1^.

Although the opportunities for women scientists are better now, there is still a long way to go before gender equality in science is fully achieved. This second edition of “*Women in psychiatry*” of the Frontiers in Psychiatry aims to promote valuable works of scientist women in the field. This edition features two studies on pharmacotherapy of psychosis spectrum disorders (PSD), including schizophrenia—one research report that discussed the long-term pharmacotherapy patterns of patients with PSD and one original research which evaluated epigenetic age and DNA methylome in patients treated with the atypical antipsychotic clozapine; one systematic review on the antidepressant escitalopram and one clinical original research article and another pre-clinical original research article, both on molecular targets of drugs of abuse.

In the study of Maric et al., “*Maintenance therapy of psychosis spectrum disorders in a real-world setting: Antipsychotics prescription patterns and long-term benzodiazepine use*,” the authors set out to shed light on the real-world prescribing pattern in PSDs in the Western Balkans, where many psychotropic medications are fully reimbursed. Guidelines to treat PSDs recommend monotherapy in detriment to polypharmacy. The authors found a high rate of antipsychotic polypharmacy (42.7%), especially in males. Benzodiazepines were the most common add-on prescribed drugs and clozapine the most prescribed antipsychotic. The study alerts about the health risks of long-term AP polypharmacy associated with BDZ.

In fact, monotherapy is usually preferred to treat PSD, with clozapine being the first-line agent in drug-resistant schizophrenia (2). In the study by Pérez-Aldana et al., “*Clozapine long-term treatment might reduce epigenetic age through hypomethylation of longevity regulatory pathways genes*,” the authors investigated epigenetic age—a biomarker of aging associated with age-related conditions and diseases—and DNA methylome in patients from Mexico treated with clozapine compared with drug-naïve patients. Clozapine induced a higher proportion of hypomethylated CpG sites in the blood compared to hypermethylated ones. Pathways enriched at hypomethylated sites included the longevity regulatory pathway, which interacts with AMPK and insulin signaling pathways. In addition, clozapine reduced the epigenetic age. Altogether, they suggested that long-term clozapine treatment might increase the life expectancy in schizophrenic patients treated with this drug.

Eichentopf et al. in “*Systematic review and meta-analysis on the therapeutic reference range for escitalopram: Blood concentrations, clinical effects and serotonin transporter occupancy*” performed a systematic review and meta-analysis on the therapeutic reference range for escitalopram (ESC), the active enantiomer of citalopram. ESC binds with high affinity to the serotonin transporter and belongs to the class of selective serotonin reuptake inhibitors (SSRI), used to treat depression, anxiety, obsessive compulsive disorder and panic attack (3). The authors investigated the association between ESC blood levels and clinical outcome—efficacy and adverse effects; ESC blood levels in relation to SERT occupancy; and factors influencing ESC blood levels. Taking into consideration the concentration/outcomes relationship, the authors suggested a target range of 20–40 ng/mL for ESC antidepressant efficacy. The review was based on 30 articles.

Substance use disorders (SUD) result in repeated relapses due to structural and functional brain alterations that persist even after periods of abstinence (4). Approved pharmacotherapies for alcohol use disorder (AUD) are limited in terms of efficacy and there are no approved treatments for stimulant dependence, justifying the need to understand the mechanisms underlying craving and relapse, and to find new and more effective therapeutic approaches.

Vamvakopoulou et al. in “*Selective D3 receptor antagonism modulates neural response during negative emotional processing in substance dependence*” investigated the effects of a dopamine D3 receptor (D3R) antagonist—GSK598809—on the neural response to negative emotional processing in individuals with AUD and SUD (cocaine/alcohol users). Blood oxygenation level-dependent (BOLD) magnetic resonance imaging (MRI) was used to assess brain function during an evocative image task, following administration of GSK598809 or placebo. The study showed that the D3R antagonist modulated relevant brain circuitry and may have restored the hypodopaminergic function observed in drug addiction (5), suggesting a potential target to further explore treatments of negative affective states in addiction.

Cue-induced drug craving is a well-known characteristic of addiction that intensifies (incubates) during protracted withdrawal, leading to relapse (6). Although extended-access intravenous drug self-administration (IV-SA) is the gold standard for modeling cocaine incubated craving, in the study by Sanchez et al., “*Profiling prefrontal cortex protein expression in rats exhibiting an incubation of cocaine craving following short-access self-administration procedures*,” simple shorter-access IV-SA also induced incubated craving. Such procedures resulted in a profile of protein expression within the prelimbic (PL) subregion of the prefrontal cortex (PFC) that is partially similar with that reported for longer cocaine IV-SA. Elevated Homer2a/b and Akt1 activation in the PL-PFC seems to be common biochemical correlates of incubated cocaine-craving across IV-SA models, highlighting a role for Akt1 signaling in the PL-PFC in the incubation of cue-induced cocaine craving. The study also demonstrated that incubated cocaine craving is associated with activated CaMKII within the PL-PFC.

## Author contributions

RC wrote the editorial. RJ and AS contributed to the review of the editorial. All editors edited the Research Topic.

## Footnotes

^1^ https://www.nobelprize.org/
